# Paired-Box Gene 8 (PAX8) and Its Association With Epithelial Carcinomas

**DOI:** 10.7759/cureus.17208

**Published:** 2021-08-16

**Authors:** Khalid Khizer, Jaskamal Padda, Anwar Khedr, Fahriba Tasnim, Ola A Al-Ewaidat, Vinay Patel, Dina Ismail, Victor Yosef Melt Campos, Gutteridge Jean-Charles

**Affiliations:** 1 Internal Medicine, JC Medical Center, Orlando, USA; 2 Internal Medicine, AdventHealth Orlando Hospital, Orlando, USA

**Keywords:** epithelial carcinoma, thyroid carcinoma, renal cell carcinoma, endometrial carcinoma, cervical carcinoma, ovarian carcinoma, thymic epithelial carcinoma, ocular epithelial carcinoma, paired-box gene 8 (pax8), paired-box gene family

## Abstract

Cancer is the second most common culprit of mortality in the United States and epithelial carcinomas are considered as one of the most predominant types of cancer. The association between epithelial cancers and paired-box gene 8 (PAX8) has been studied significantly before. PAX8 belongs to the paired-box gene family, which plays an important role in the organogenesis of different body organ systems, especially the thyroid gland, the renal system, and the Müllerian system. Immunohistochemical staining is being used to detect PAX8 expression in different epithelial cancers and differentiate them from PAX8-negative tumors. In follicular, papillary, and anaplastic thyroid carcinomas, targeting the PAX8/peroxisome proliferator-activated receptors (PPARs) fusion protein is being considered as a potential mechanism for therapy. Moreover, because of its high expression in primary ovarian cancers, PAX8 is being considered as a target for ovarian cancer treatment as well. More studies are needed to test the possibility of using PAX8 as a possible target for managing endometrial carcinomas. In this article, we review the functions of the PAX8 gene, how its mutations lead to the development of certain epithelial carcinomas, how it can be used as a diagnostic or a prognostic marker, and its potential as a therapeutic target for these cancers.

## Introduction and background

Cancer is the second leading cause of death in the United States [1]. In 2021, 1,898,160 new cancer cases and 608,570 cancer-related deaths are estimated to take place within the United States [2]. Most of these neoplasms are of an epithelial origin, which arises from the tissues that line all body cavities [3]. Due to this dreadful condition, it has always been a priority to detect cancer at its earliest phase. Although there are many cancer screening and detection methods, it sometimes gets challenging to identify the primary cancer site once it metastasizes. In this situation, PAX8 immunostaining has become a ray of hope.

PAX8 is a member of the paired-box family of genes that plays a crucial role in the development of the kidney, thyroid gland, and Müllerian organs [4-6]. PAX8 positivity has also been demonstrated in several cases of renal, thyroid, and ovarian tumors [7-12]. Due to its high sensitivity and absence of expression in breast, lung, gastrointestinal, and mesothelial malignancies, PAX8 immunohistochemistry is now routinely used as an adjunctive tool in diagnosing tumors, especially when they become metastatic [10, 12-14].

In this review, we discuss the function and pathophysiology of the PAX8 gene. We also shed some light on the role of PAX8 in the development of various cancers, such as thyroid, renal, ovarian, endometrial, cervical, and ocular, as well as thymic epithelial carcinomas. We will also discuss the significance of PAX8 immunostaining in detecting these cancers.

## Review

Definition and function of PAX8

Genes of the paired-box family encode transcription factors containing the deoxyribonucleic acid (DNA)-binding paired-box domain. They have been further subclassified into four groups (I-IV) based on the presence of an octapeptide region, paired-type homeodomain, or both [6, 15]. These transcription factors play a vital role in organogenesis during embryonic development and prevent functional abnormalities in some cells after birth. The PAX8 gene was mainly found to have a vital role in the thyroid gland and kidney embryogenesis [13]. It regulates the differentiation of renal and thyroid follicular cells and controls thyroid hormone release.

Moreover, it has a critical role in the urogenital system morphogenesis, as well as the organogenesis of the central nervous system, eye, mesonephric (Wolffian), and Müllerian ducts [16]. In addition, it was found to be expressed in the excretory system of the kidneys, endocervical epithelial cells, uterine endometrium, ovaries, fallopian tubes, seminal vesicles, epididymis, lymphoid cells, and islet cells of the pancreas [17].

Pathophysiology of PAX8

Congenital Hypothyroidism

A loss-of-function mutation in the PAX8 gene was found to cause congenital hypothyroidism. These mutations limit the ability of the PAX8 gene to bind to DNA and interact with other transcription factors. This interferes with the thyroid gland's normal development and function during embryonic life, resulting in thyroid dysgenesis and abnormally low thyroid hormone levels beginning at birth [18-19].

Tumors

Although the PAX8 transcription factor is crucial in the survival of the differentiated epithelial cells, its expression level can modify the growth rate of these cells. Therefore, it was found that overexpression of this gene under abnormal conditions is related to the development of different tumor types [20]. The PAX8 gene was found to be overexpressed in different tumor types, including thyroid and renal carcinomas, ovarian epithelial carcinomas, and pancreatic neuroendocrine tumors [21]. Based on this, the PAX8 gene can be used for screening and differential diagnosis of these tumors.

Role of PAX8 in thyroid carcinomas

Thyroid cancers are usually well-differentiated and associated with less mortality. Papillary thyroid cancers are the most prevalent subtype in general. Hürthle cell cancers (HCCs) and follicular thyroid cancer (FTC) together account for 20% of all thyroid cancers. The latter thyroid cancer types are more dangerous, more frequently discovered in advanced stages, and less responsive to treatment [22].

Peroxisome proliferator-activated receptors (PPARs) are recognized for their role in insulin sensitization and adipogenesis. PPARs, through influence on gene expression of cell signaling pathways, play an essential role in cell cycle control and carcinogenesis [23]. In a subset of FTCs, PPAR translocation has been discovered to play a role as a thyroid protooncogene in some studies [22, 24]. PAX8/PPAR fusion protein (PPFP) is a fusion protein expressed from a neogene which is made from translocation, and it functions to bring PPAR and PAX8 genes together. PPFP has been seen frequently in FTCs [24]. PPFP’s impact on PAX8 function has been assessed in a few studies [22]. PAX8 regulates genes for sodium iodide symporter (NIS), thyroid-stimulating hormone receptor (TSHR), thyroid peroxidase (TPO), and thyroglobulin (Tg) promotors. PPFP’s effect on PAX8 seems complex; PPFP downregulates the expression of TSHRs and Tg while it upregulates the expression of NIS and TPO [22].

Anaplastic carcinoma can be difficult to differentiate from sarcoma or squamous cell carcinoma (SCC) due to poor immunohistochemistry and morphology. PAX8 is expressed in normal and neoplastic thyroid cells. A study showed PAX8 expression in 76% of 34 patients with anaplastic carcinoma [25]. In the same study, all the head and neck squamous cell carcinomas had negative results for PAX8. Thus, PAX8 proved to be an excellent marker for follicular epithelial origin, even poorly differentiated ones, to make the differential diagnosis from head and neck squamous cell carcinoma.

Various studies, using reverse transcription-polymerase chain reaction, fluorescence in situ hybridization, nested polymerase chain reaction, or western blot analysis, have pointed out a higher prevalence of expression of PPFP in FTCs [22]. Precise measurements, however, are dependent on study methods and scale. A 36% incidence has been noted in FTCs and 11% rates in follicular adenomas. The PAX8/PPAR rearrangement has only been seen in one case of HCC. The papillary variant of FTCs, along with very few cases of HCC, has also shown PPFP expression [26].

Data suggests PPFP can alter pathways which, in turn, stimulates cell proliferation, leading to tumorigenesis. It has also been shown to alter targets of current therapeutic models through PPAR inhibition or PAX8 expression modulation mechanisms. This is why PPFP can be a target in various thyroid cancer treatments, like follicular, anaplastic, and papillary thyroid cancers [22].

Role of PAX8 in renal cell carcinoma

PAX8 plays a role in the regulation of renal organogenesis. Additionally, it is highly expressed in renal epithelial cells of the adult kidney. PAX8 was discovered to be an oncogene in renal cell carcinoma (RCC) cells by Bleu et al. [27]. They discovered that the transcription factor produced by this gene stimulates the expression of metabolic genes, such as ferroxidase ceruloplasmin, by binding to distal enhancer sites. Ceruloplasmin expression has been related to PAX8 silencing sensitivity in cell lines. Additionally, an inverse association between survival among RCC cases and expression of ceruloplasmin was established. Moreover, there is decreased proliferation of RCC cell lines because of PAX8 silencing. Further analysis revealed that several genes linked to the cell cycle and metabolic processes are also regulated by PAX8. Due to these facts, PAX8 has the potential to play a crucial role in RCC development [27]. PAX8 can be detected in all segments of the renal tubules, from the proximal tubules to the renal papillae as well as in the parietal cells of Bowman’s capsule [7]. Several studies have also shown that PAX8 can be detected in samples of different types of RCC which are summarized in Table 1 [7, 13, 28-30].

**Table 1:** Summary of the Studies Reporting the Percentage of PAX8 Expression in Renal Cell Carcinoma NA: not available; RCC: renal cell carcinoma

	Clear Cell RCC	Papillary RCC	Chromophobe RCC	Renal Oncocytoma
Tong et al., 2009 [7]	98% (58/59)	90% (19/21)	82% (9/11)	95% (21/22)
Barr et al., 2015 [28]	80% (165/207)	95% (39/41)	100% (6/6)	NA
Hu et al., 2012 [29]	93% (78/84)	95% (54/57)	80% (53/66)	94% (15/16)
Tacha et al., 2011 [30]	91% (86/94)	100% (14/14)	57% (4/7)	NA
Laury et al., 2011 [13]	93% (115/123)	76% (19/25)	80% (4/5)	81% (13/16)

Furthermore, Ozcan et al. reported the frequency of PAX8 expression to be 82% - 89% among American patients with RCC [31]. Knoepp et al. also noted the frequency to be 88% among American patients [32].

Due to the high expression of PAX8 in RCC, it can be used for screening as well as risk stratification [32]. Also, it can be used as a diagnostic tool that can play a supportive role in diagnosing RCC, alongside core biopsy, as it can be challenging to obtain a sample from metastatic lesions. About 5% of primary tumors are considered unclassified, even after resection, due to poor differentiation or a mixed morphological pattern [28]. Also, metastatic tumors arising from the kidney may resemble other malignancies as they may have different characteristics than the primary tumor or may be poorly differentiated [33]. Thus, it may be challenging to detect tumors in advanced stages. For these reasons, pathologists are inclining more towards the use of immunohistochemistry in diagnosing primary and metastatic RCC to distinguish it from other primary tumors. A survey conducted by the International Society of Urologic Pathologists found that about 90% of pathologists use immunohistochemistry to confirm the diagnosis of metastatic RCC [34]. While many pathologists reported using various markers, such as cytokeratin 7 (CK7), vimentin, and a cluster of differentiation 10 (CD10), most of them stated PAX2 and PAX8 to be the most valuable markers. However, in another study, PAX8 was found to be more sensitive than PAX2 and considered eligible to play an essential role in the diagnosis of primary renal neoplasm [35].

Role of PAX8 in endometrial carcinoma

PAX8 has also been associated with certain endometrial cancers due to its role in cell growth. Therefore, a study on 229 patients was done to find the correlation of PAX8 expression in specific clinical parameters of endometrial cancer [36]. Positive PAX8 results were correlated with high tumor grade (P = 0.002), type 2 tumor subtype (P < 0.0001), and lymphovascular invasion (P = 0.0186). In addition, the five-year survival with a disease-free state was calculated as 49.88% (P = 0.02028) in PAX8-positive patients compared to 72.12% in PAX8-negative patients. Also, PAX8-negative patients were associated with 74.36% chances of recurrence-free survival compared to 52.11% in PAX8-positive.

In another study, 106 patients with primary endometrial carcinoma were studied over a 10-year period, 97 of which were investigated further based on eligibility [37]. P53 and PAX8 expressions were calculated using an immunohistochemical technique and data was clinically correlated. PAX8 was found to be positive in 72.1% of 97 patients, 72.7% of 77 endometrial cancer patients, and 72.2% of 18 patients with non-endometrioid cancers. Histological type, grade, and p53 expression data also showed a positive correlation. Vessel space involvement, lymph node involvement, stage of the tumor, and age were also assessed, with no correlation with PAX8. Patient survival was also shown to have no statistically significant correlation with PAX8 with a five-year survival rate of 76% in PAX8-positive patients compared to 72% in PAX8-negative patients.

In yet another study, 52 endometrioid endometrial cancers, 21 human papillomavirus-related endocervical cancers, 21 serous endometrial cancers, 11 benign mesonephric growths, and 58 normal endometrium patients were studied for PAX8 expression [38]. Both serous and endometrioid endometrial cancers were shown to have a high association with serous endometrial cancer, expressing significantly high levels of PAX8. Endocervical adenocarcinomas were also associated with a high frequency of PAX8 expression but not as much as endometrial adenocarcinomas. High PAX8 expression in such various types of intrauterine malignancies makes it less useful as a diagnostic marker. Significantly low expression of PAX8 was noted with the normal or benign proliferation of the endocervix and endometrium patients. However, in extrauterine sites, it can be a valuable marker for adenocarcinomas of uterine origin. The sensitivity of identifying metastasis is less and depends upon tumor cells in the metastatic site [38]. In general, PAX8 can be considered a poor prognostic marker in patients with endometrial carcinomas and can be targeted for therapy in selected patients, the utility of which needs more clinical data [36].

Role of PAX8 in cervical carcinoma

PAX8 is a strictly regulated transcription factor that has also shown increased immunostaining in cervical carcinomas. As PAX8 plays an essential role in the embryogenesis of the Müllerian ducts, PAX8 expression is noted in carcinomas developed from this region. Although the PAX8 gene gives rise to four isoforms through alternative messenger ribonucleic acid (mRNA) splicing, there is no available data that can determine which PAX8 isoform is present in cervical tissues and cervical carcinoma. Instead, numerous previously unreported PAX8 aberrant transcripts were found in both cervical carcinoma-derived cell lines and tumor samples [39].

On a separate note, it has been established that the E2F transcription factor 1 (E2F1) promoter is transcriptionally regulated by PAX8 directly, and RB1 depletion enhances E2F1 transcription leading to persistent cell growth [40]. When a cell acquires TP53 or RB1 mutation, it may generate epithelial lesions, such as endometrial cancer [41]. Similarly, we can detect PAX8 upregulations in cervical cells infected with human papillomavirus (HPV) as viral proteins disrupt TP53 and RB1 functions. In a recent study, RNA analysis in cervical samples uncovered upregulation of PAX8 transcripts in HPV-positive lesions, presumed to be the main culprit behind cervical carcinoma [42]. Several studies reported PAX8 expression in adenocarcinoma, SCC, endometrioid adenocarcinoma, and adenosquamous carcinoma of the uterine cervix [13, 30-31, 38, 43-51].

Shukla et al. reported PAX8 immunostaining in 64 of 66 cases of adenocarcinoma in situ and 11 of 55 cases with high-grade squamous intraepithelial lesions of the cervix [43]. A case report noted that PAX8 immunostaining was valuable in diagnosing metastatic cervical cancer of the breast [44]. Several other studies demonstrating PAX8 immunostaining in cervical cancers are summarized in Table 2 [13, 30-31, 38, 45-51].

**Table 2:** Summary of the Studies Reporting the Percentage of PAX8 Expression in Cervical Carcinomas NA: not available; SCC: squamous cell carcinoma

	Adenocarcinoma	SCC	Endometrioid Adenocarcinoma	Adenosquamous Carcinoma
Laury et al., 2011 [13]	1/2	2/2	NA	NA
Tacha et al., 2011 [30]	5/6	1/60	NA	1/3
Tong et al., 2011 [45]	0/5	NA	NA	NA
Woodard et al., 2011 [46]	12/19	NA	6/9	NA
Danialan et al., 2013 [47]	2/12	NA	NA	NA
Yemelyanova et al., 2014 [38]	18/21	NA	NA	NA
Goyal et al., 2014 [48]	13/15	NA	NA	NA
Liang et al., 2016 [49]	35/43	NA	NA	NA
Ozcan et al., 2011 [31]	NA	0/9	NA	NA
Gailey et al., 2013 [50]	NA	3/11	NA	NA
Wong et al., 2017 [51]	14/20	8/103	5/6	2/7

Although several specimens expressed PAX8 immunostaining in cervical cancer samples in several studies, they were not highly specific [13, 30-31, 38, 45-51]. From the aforementioned studies, we can see that most SCC and a significant number of adenocarcinomas of the cervix lacked PAX8 staining. Hence, PAX8 immunostaining can play an essential role as an adjunct tool in diagnosing cervical cancer, but a negative result cannot rule out the diagnosis [51]. Furthermore, the usefulness of PAX8 staining in differentiating endocervical lesions from endometrial lesions is limited [52].

Role of PAX8 in ovarian carcinoma

Ovarian cancer has the most unfavorable prognosis of all cancers of the female reproductive system. This is due to its subtle presentation and the absence of reliable screening and diagnostic tests which makes early detection of this disease very challenging. The high-grade serous carcinoma subtype is the most prevalent and severe type of ovarian cancer [53]. The PAX8 gene is expressed in the adult fallopian tube epithelial cells but not in the ovarian epithelial cells. However, it was found that PAX8 gene expression was clearly detected in ovarian epithelial cancer cells. This indicates that the origin of these cancer cells is from the fallopian tube epithelial cells [54].

The exact role of the PAX8 gene in ovarian epithelial cell carcinogenesis is still unclear. However, the PAX8 gene was found to play an essential role in the motility, adhesion, invasion, and tumorigenesis of ovarian cancer cells by modulating these cells' interaction with the extracellular matrix. This characterizes the metastatic way of this cancer, which is direct seeding and invasion of the peritoneal cavity [55].

The current use of cancer antigen (CA)-125, estrogen receptor, and progesterone receptor for the diagnosis of ovarian cancer are unreliable due to their low specificity and sensitivity. This further complicates the early detection of this disease [56-57]. Based on the immunohistochemical studies, the PAX8 gene was clearly expressed in the primary ovarian cancer cells but not in the metastatic ovarian cancer cells, which indicates the high specificity of this gene expression in primary ovarian cancer cells. Therefore, the PAX8 gene is being considered as a potential effective diagnostic marker of ovarian cancer. Moreover, it was discovered that the level of this gene expression is proportional to the degree of differentiation of cancer cells. It was found that higher PAX8 gene expression was associated with a lower cancer cell differentiation and lower survival rate. This gives PAX8 gene the advantage of being a prognostic factor of ovarian cancer as well [57].

Due to the enormous diversity of ovarian cancer cell mutations, it has been challenging to find targets for the effective treatment of this disease. However, the PAX8 protein is regarded to be a promising target for ovarian cancer treatment based on its pervasive expression in these cancer cells [54]. This gives hope to decrease this aggressive tumor's burden and progression and further increase the patient's survival.

Role of PAX8 in thymic epithelial carcinoma

Neuroendocrine cancers of the lung and neuroendocrine tumors from the thymus have similar clinical presentations, although they are very different in their biological behavior. Studies have shown a significantly high level of mortality associated with the atypical origin of neuroendocrine tumors [58-59]. The five-year survival of thymus origin carcinoid tumors was calculated to be 50% compared to atypical carcinoid tumors having a low rate of 5%. This makes the differential of these two based on origin very important. PAX8 has been drawing attention as a potential marker. PAX8 is expressed in epithelial tumors in different organs, as well as in thymic epithelial neoplasms. According to many studies, pulmonary neuroendocrine cancers have failed to express PAX 8. Thus, PAX8 can be used as an immunohistochemical marker that can be used to differentiate lung from thymic origin tumors [60]. In yet another study, PAX8 immunoreactivity was assessed in 31 thymic carcinomas, 30 patients with World Health Organization (WHO) type B thymoma, and 30 patients with WHO type A thymomas [61]. Seventy-seven percent of thymic cancers, 93% of WHO type B thymomas, and 100% of WHO type A thymomas showed positive PAX8 immunoreactivity. In another small study on 13 thymic cancer cases and 15 poorly differentiated lung cancer cases, patients were analyzed for PAX8 expression [62]. In that study, 69.2% of thymus origin cancers showed PAX8-positive results compared to 5.8% in lung cancer patients. The study concluded that PAX8 had a high diagnostic value in patients with thymic cancers.

Role of PAX8 in ocular epithelial carcinoma

Intraocular metastasis of malignant tumors represents the most common intraocular tumor. However, retinoblastoma and malignant uveal melanoma are the most common primary intraocular tumors [63]. The intraocular expression of the PAX8 gene was observed in the retinal neurons, the iris sphincter pupillae muscle, and the dilator muscle complex. It was also found to be expressed in the epithelium of the cornea, lens, iris pigment, and ciliary body. A subset of intraocular tumors demonstrated a clear PAX8 gene expression. This includes epithelial and neuroepithelial ciliary body tumors, retinoblastoma, and uveal melanoma. However, its expression was not detected in all melanocytic and retinal pigment epithelial tumors. Therefore, the PAX8 gene is being considered as a target of an effective diagnostic marker of different intraocular tumors [64-65]. Although PAX8 is expressed in the lens epithelium, it was found to be absent in the lens epithelium with mesenchymal transition, including capsular fibrosis and subcapsular cataract [66]. Based on that, it does not represent a reliable diagnostic marker for these lesions.

## Conclusions

The object of this review is to examine the association between PAX8 and epithelial carcinomas. PPAR translocation and fusion with PAX8 was found to play a role in the pathogenesis of some types of follicular thyroid carcinoma. PAX8 is being evaluated by pathologists using immunohistochemistry to diagnose primary and metastatic RCC. PAX8 is also being used to differentiate primary ovarian carcinoma from metastatic carcinomas and as an additional way to confirm the diagnosis of cervical carcinomas. Moreover, PAX8 is utilized to differentiate thymic epithelial neoplasms from other pulmonary and mediastinal epithelial masses. Intraocular ciliary body epithelial neoplasms were found to be PAX8-positive; this is why it is considered a valuable tool to differentiate these tumors from other intraocular melanocytic tumors. In addition, PAX8 expression is regarded as a poor prognostic factor in cervical and endometrial epithelial carcinomas. The role of PAX8 as a treatment target has not been thoroughly studied yet, so more studies are needed to evaluate the potential of targeting PAX8 to treat different PAX8 positive epithelial carcinomas, especially thyroid, ovarian, and endometrial carcinomas.
